# Electrochemical Sensors as a Tool for Taste Perception in Pharmaceutical Products: Advances and Perspectives

**DOI:** 10.3390/bios16020084

**Published:** 2026-01-30

**Authors:** Juliana Luz Melo Gongoni, Marilia Medeiros, Hatylas Azevedo, Margarete Moreno de Araújo

**Affiliations:** Innovatech Laboratory, Aché Laboratórios Farmacêuticos, Rodovia Presidente Dutra—Porto da Igreja, Guarulhos 07034-904, SP, Brazilmarilia.medeiros@ache.com.br (M.M.); hatylas.azevedo@ache.com.br (H.A.)

**Keywords:** electronic tongue, e-tongue, taste-masking, pharmaceutical, sensory evaluation, taste evaluation, data processing, multivariate data

## Abstract

Taste masking in pharmaceutical products is a complex and subjective process that requires reliable evaluation methods. This review focuses on the electronic tongue (e-tongue), an emerging sensor-based technology designed to mimic human taste perception without the need for human panels. E-tongue systems provide objective data to support the development of palatable formulations. In this review, we discuss the principles, types of e-tongue devices, data processing approaches, and their applications in pharmaceutical research. By comparing e-tongue performance with human taste assessment, we highlight its potential as a complementary tool to traditional in vitro assays, accelerating formulation development and improving patient adherence.

## 1. Introduction

Human survival relies on food, and this connection is intrinsic to our existence. Initially, the relationship with food, similar to that of other animals, depended on taste and smell [1]. As the population has grown and the subsequent demand for food has increased, the taste-related industry experienced rapid expansion [2].

This extends to the pharmaceutical industry, where the taste of active pharmaceutical ingredients (APIs), often bitter, presents a significant challenge [3]. Taste-masking is crucial to palatability, mainly due to the unpleasant taste of some APIs. The palatability of medicines can directly affect patient adherence, especially in pediatric and geriatric populations [4]. Poor adherence is particularly prevalent among these groups and can be influenced by the flavor of the medication [5].

Large pills are impractical for children due to their inability to swallow them, necessitating liquid or orally dispersible formulations that are effective and safe, considering differences in pharmacokinetics and excipients when compared with those of adults. For elderly patients, larger-sized pills, combined with conditions like dysphagia and use of numerous medicines (polypharmacy), make solid oral dosage forms unsuitable [6]. In these cases, liquid or dispersible formulations that are easy to swallow, visually appealing, and palatable are more appropriate for these patients [7,8].

These challenges have also driven the exploration of taste as a scientific field. Traditional taste evaluation relies on trained human panels, making it subjective and leading to variability due to contextual factors [9]. Each panelist may perceive taste, texture, or mouthfeel differently based on factors such as age, gender, cultural background, prior experiences, and even genetic differences in taste receptors. Additionally, psychological factors and expectations can influence how a product is perceived. As a result, the data obtained may lack consistency and reproducibility, making them less reliable for objective comparison across formulations or studies [10].

Gold-standard analytical methods like HPLC, GC, and mass spectrometry offer precision and real-time monitoring. However, they are costly, require trained personnel, and can only evaluate molecular fingerprints compared to a data bank, not the subjectivity of taste likability [11].

An alternative lies in sensor-based technologies, which can provide real-time data [12]. Among these sensors are electronic sensing systems, pioneered with the electronic nose in 1964 by Wilkens and Hartman [13] and later followed by the electronic tongue in the 1990s, introduced by Vlaslov [14] and defined by Toko [15].

Electronic tongues utilize sensors that target specific compounds or provide a generalized analysis, such as pH, ionic strength, or concentration [12]. These sensors could be tailored for taste sensing to detect specific taste profiles (bitter, sweet, sour, salty, or umami) or employ cross-selectiveness to acquire a comprehensive sample profile.

The electronic tongue offers notable advantages, including precision and objectivity compared to human panels and a reduced sample number for sensory testing. E-tongues streamline the process, retaining only the most promising formulations for further evaluation. Moreover, it could save time and resources [12].

**Figure 1:**
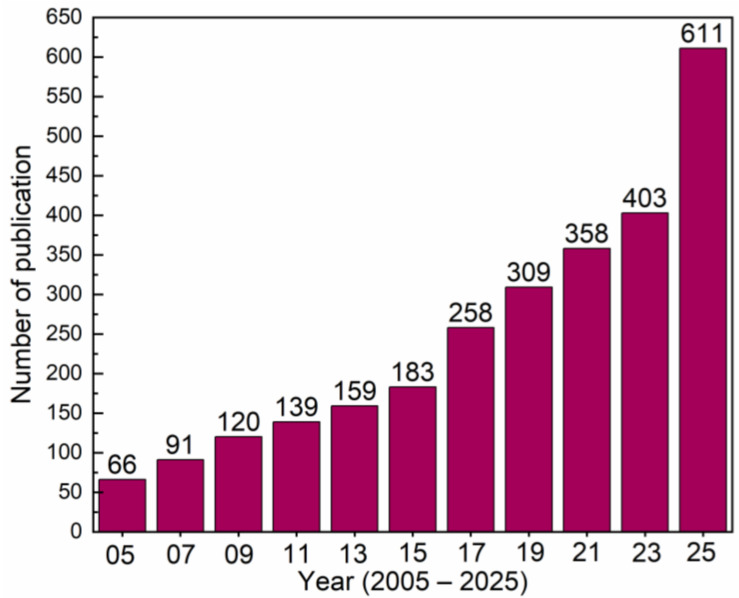
Number of papers published in the last 20 years. The information was extracted from the Web of Science using the keywords “electronic tongue” or “e-tongue”. The data were obtained up to December 2025.

Interest in this technology continues to grow, as evidenced by the increase in publications referring to the “electronic tongue” or “e-tongue” in the Web of Science database. The field remains vibrant, evolving from just one paper in 1947 to 973 papers in 2024, and in the last decade, 11,248 papers were published up to May 2025 (Figure 1).

This review comprehensively analyzes how taste perception works in the human tongue and how an electronic tongue can be used for taste evaluation in the pharmaceutical industry. Numerous topics about electronic tongues will be explored, such as history, different types of electronic tongues, a review of sample applications from the literature, data processing methods, comparisons with other methodologies, taste masking, and a highlighting of their potential in pharmaceutical industry applications.

## 2. Human Tongue

The human tongue served as inspiration for the development of the electronic tongue. The tongue is a movable group of muscles in an oblong shape, consisting of connective tissue and nerves [16]. This muscle organ can perform various functions, including sucking, swallowing, speaking, helping protect against pathogens, feeling, and tasting [16]. The latter two are essential components of the sense of taste, as the tongue’s properties enable the evaluation of food, determining if it is fresh, spoiled, toxic, or safe to consume [17].

It was mistakenly believed that the five tastes were recognized in specific tongue regions. Taste perception is mediated by taste buds, which are responsible for this process. Sömmerring provided the first accurate tongue description in 1806 [18], describing that the tongue’s surface consists of a mucous membrane containing taste buds located within the papillae, which can be divided into various forms as follows:

- Vallate papillae (or circumvallate papillae) are found at the back of the tongue in a V-shaped arrangement. A person can have between four and eighteen of these visible structures [19].
- Fungiform papillae are pink, round, and scattered across the tongue, increasing in size from the tip to the back and are found on the sides [19,20]. Taste buds are located on the top of these papillae.
- Filiform papillae cover most of the dorsal surface of the tongue. These narrow, conical structures are made of keratin, giving the tongue its texture, hairy appearance [21]. Unlike other papillae, filiform papillae do not contain taste buds and instead help with chewing [21].
- Foliate papillae are located exclusively on the sides of the tongue, forming vertical ridges with taste buds situated between the grooves [22].

**Figure 2:**
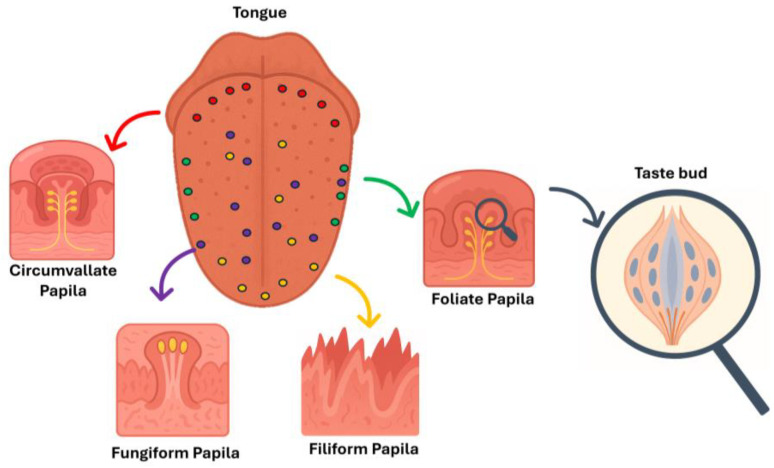
Schematic drawings of the tongue showing the representation and overall location of each papilla type. The magnifying glass shows a representation of a taste bud. The image was created with the aid of ChatGPT-5, OpenAI, 2025.

Papillary structures are shown in Figure 2. Additionally, taste buds are present in other areas, such as the soft palate, pharynx, larynx, and esophagus [23].

Taste buds themselves are bulb-shaped structures composed of 50 to 120 slender cells, as represented in Figure 2. These cells are classified into five types (I to V):

- Type I cells, which account for 50% of the total, are glia-like and spindle-shaped. They are involved in the transduction of salty taste [24].
- Type II cells, located at the periphery of the taste bud, detect sweet, bitter, and umami tastes through TAS1R and TAS2R family receptors.
- Type III cells, representing 5–7% of the total, form synaptic contacts with nerve fibers and are referred to as “presynaptic cells”. They sense sour tastes via channels in their taste receptor cells (TRCs) [25].
- Type IV cells are undifferentiated progenitor cells that give rise to other cell types.
- Type V cells are marginal cells [19].

Taste can be felt when food or medicine dissolves into the saliva, allowing molecules to interact with specific receptors on taste buds. These interactions trigger recognition signals that vary in specificity [26], as some receptors can detect multiple molecules (known as promiscuous), while others are highly selective [27,28]. Signals are then transmitted via cranial nerves (facial, glossopharyngeal, or vagus) to the brain, where final taste perception occurs [29].

Taste is a complex process closely linked to the olfactory system. While taste buds can detect five basic tastes (sweet, sour, salty, bitter, and umami), finer distinctions rely on retro-nasal smell pathways during chewing and swallowing [29,30,31,32,33]. The final processing occurs in the cortical region of the brain. Interestingly, a recent report by Sternini et al. (2024) [26] found mRNA expression for bitter taste receptors throughout the gastrointestinal tract, suggesting these receptors may also play a role in digestion.

The number of taste buds changes throughout life. Infants have around 10000 taste buds, but this number declines after the age of 50 to 60 years. Each taste bud has a lifespan of about 10 days [23]. Taste preferences also evolve in infants and children, who prefer sweet tastes, while adolescents and adults are more open to bitterness, sourness, and savory foods. In older adults, taste preferences may shift due to diminished perception, often leading to a preference for sweet or more pungent foods [29,33].

Given the subjectivity of taste perception, it can be challenging to assess taste consistently. Trained personnel must ensure accurate evaluations with minimal variance. This task can be costly, especially when APIs require hospitalization, medication-specific panels, or strict safety protocols. As a result, electronic sensors, such as the electronic tongue, have been developed to assist in information selection or to reduce the number of samples needed for human panel testing.

## 3. Electronic Tongue

### 3.1. Electronic Taste System

An electronic tongue (or e-tongue) is equipment designed to identify taste without relying on in vivo experimentation. Essentially, an e-tongue constitutes an electrode array submerged in a liquid medium capable of analyzing complex matrices and providing a unique fingerprint of the sample. According to the International Union of Pure and Applied Chemistry (IUPAC), an e-tongue is defined as a device that combines an array of low-selective sensors with multivariate analysis or pattern recognition techniques [14].

This innovative technology has gained significant attention, especially in industrial applications, as a viable alternative to flavor analysis across various sample types [34,35]. Additionally, the e-tongue offers a user-friendly and cost-effective solution, enabling the execution of rapid and straightforward analytical protocols [36].

The idea of electronic evaluation, before any electronic tongue, began in 1943 with McCulloch and Pitts et al. [37] with the idea of mimicking the brain. They developed a model that proposed computational elements based on the physiological properties of a biological neuron and its connections, thus introducing the foundational reference for the theory of artificial neural networks. The proposal was to perform through mathematical algorithms the brain’s synapses to be able to predict sensations and conclusions. For this aim, electronic sensors were researched, each corresponding to an individual sensation [14].

The first to be reported in the literature was an electronic nose prototype (or e-nose for short) in 1964, pioneered by Wilkens and Hartmann [13]. They had already reported in 1954 [38] the olfactory sensation through electronic analogs and in 1957 [39] the electronic instrumentation necessary to construct such sensors. The e-nose was an array of microelectrodes that were able to sense a variety of gases [13,40]. A fully complete e-nose was designed in 1982. In 1989, the first patent that mentions a taste sensor was filed by Toko and collaborators [41]. The first mention in the literature was in 1990 when the membrane constitution, working basics, and first responses were reported. This led to a collaboration between Toko, represented by Kyushu University, with Anritsu Corporation that generated the first taste sensor, SA401 [42], in 1993. It was not until 1995, when the taste sensor would be called an electronic tongue [41,43], that it was considered a device for quantitative as well as qualitative analysis [14]. In 1996, Anritsu released SA402 [42], a more integrated and compact system, with a user interface that was compatible with only Windows 95 and closer to the system by which it is better known nowadays.

It is worth mentioning that all the e-tongues until 1997 were potentiometric (definition of each analysis method will be discussed below). Winquist and Lundstrom [44] developed the first voltammetric e-tongue that allowed a reduction in noise, with more forms of voltammetry (cyclic, stripping, and pulse) as well as sensor materials. However, it may lead to convoluted signals.

**Figure 3:**
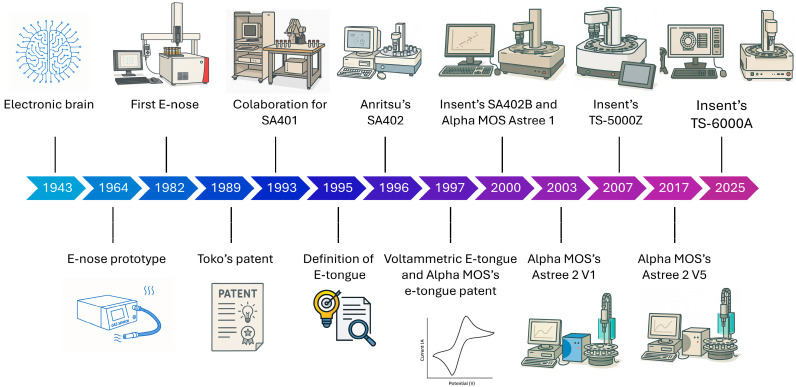
Electronic tongue storyline. The image was made with the aid of ChatGPT-5, OpenAI, 2025.

Moreover, in 1997, Alpha MOS submitted their patent regarding e-tongue development [45]. In 2000, InSent SA402B was released, with more compatible operating systems, Windows 98, ME, 2000, XP, and 7, as well as IBM PC/AT-compatible computers [42], and Alpha MOS with Astree 1 [46]. In 2007, InSent (former Kyushu and Anritsu collaboration) launched the TS-5000Z, a more modern presentation with internet communication, associated with a Linux server. From 2003 up to 2016, Astree 2 V1 to V4 were released on the market. In 2017, V5 was designed and remains available nowadays. In 2025, a new model of InSent’s equipment, TS-6000A, was introduced with a better interface, a fully integrated monitor, and barcodes integrated into the operating system. The timeline of the electronic tongue systems’ emergence can be seen in Figure 3, and the summarized information can be found in Table 1.

**Figure 4:**
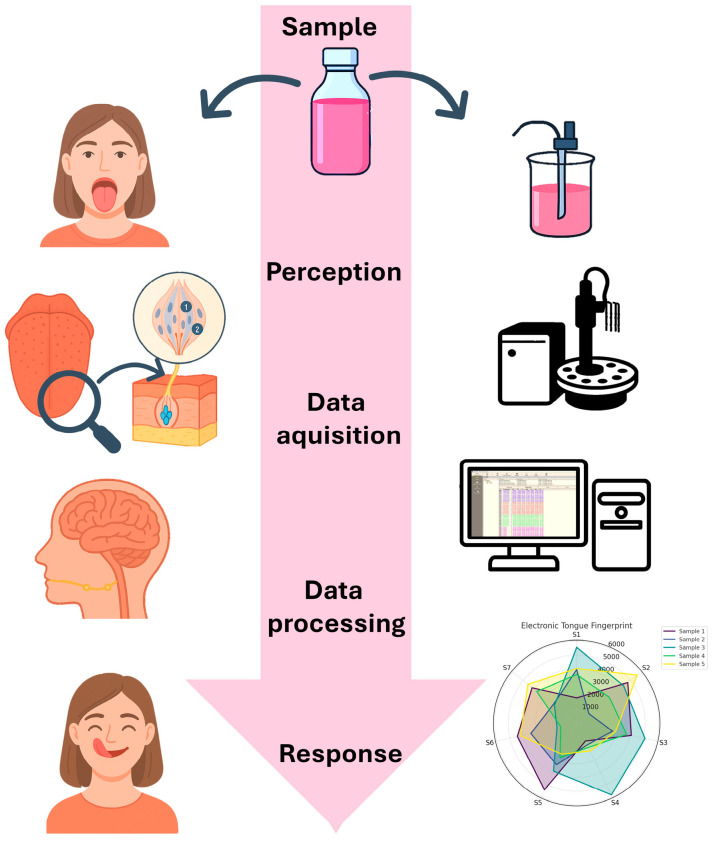
Scheme comparing how an electronic tongue and a human tongue work. The image was made with the aid of ChatGPT-5, OpenAI, 2025.

An electrochemical e-tongue is constituted of an array of electrodes, which can either be unspecific and capable of sensing various molecules, or equipped with specialized membranes to target specific tastes. In commercial setups, these sensors may be assembled on a mechanical arm for ease of use. The system also includes a data acquisition unit and amplifier (potentiostat), a data processor, and a computer for data treatment [47]. This configuration mimics the functioning of the human tongue, as schematically represented in Figure 4.

Unspecific sensors can mimic the human tongue by detecting the five basic tastes using multiple sensors or analyzing a molecular fingerprint. These electrodes are usually made of chalcogenide glasses [48] or noble metals to recognize various molecules. However, this approach can generate convoluted signals, due to a broader electrode that is not selective. To address this issue, membranes can be incorporated to target specific analytes as reported by Iiyama et al. [49]. These membranes, typically made of lipids/polymers, transform chemical information into electric signals by altering membrane potential [50,51].

These membranes can be tailor-made to detect a single taste. They exhibit higher sensitivity and durability than human taste buds, enabling the detection of lower concentrations and extending their functional lifetime. Common lipid materials include oleic acid, cholesterol, dioctyl phosphate, decyl alcohol, 1-hexadecanol, gallic acid, and many other possibilities [15,52]. Plasticizers such as trioctyl trimellitate, dioctyl phenylphosphonate, 2-nitrophenyl octyl ether, bis(1-butylpentyl) adipate, and others can also be used, and the polymer commonly used is PVC [15,41,53]. The electrodes with these membranes serve as working electrodes, complemented by an Ag/AgCl/KCl reference electrode.

**Figure 5:**
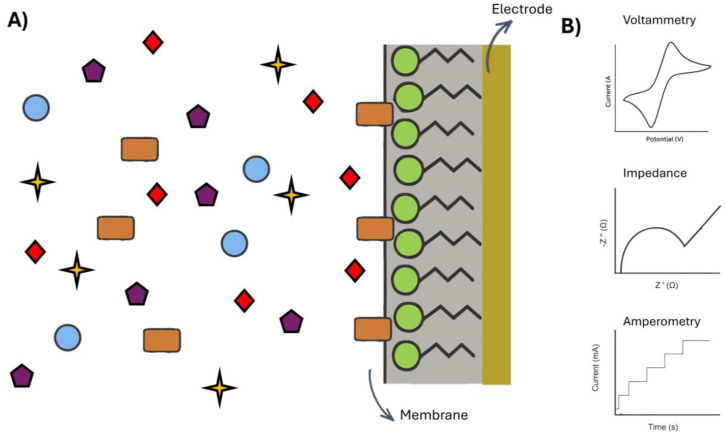
(**A**) Schematic representation of a membrane sensor with different forms of detection. (**B**) Representation of the main electrochemical techniques used with an e-tongue. The different shapes in the figure represents different molecules in the solutuion. The image was made with the aid of ChatGPT-5, OpenAI, 2025.

E-tongues can operate in various modes, with potentiometric [51,54,55,56] and voltammetric [57,58,59] being the most common. Other methods, such as impedimetric [60,61] and amperometric [62], are also used, as represented in Figure 5.

Voltammetry is a technique that constitutes applying a potential, and, as a result, a current flows between the electrodes, which is recorded during the measurement. The information about the analyte is obtained through the potential change and current acquisition. The voltammetric response not only depends on the analyte concentration but also on the diffusion layer. The diffusion layer is created by the analyte coming from the solution bulk to the electrode’s surface, creating a concentration gradient. How fast or slow this process occurs is the response seen in the voltammogram graph. The analyte migration not only depends on the intrinsic properties but also on the sweep rate. The sweep rate, also known as scan rate, is how fast the potential variation is applied in a certain amount of time. This implies that slower sweep rates obtain more detailed information, and therefore, more defined processes. On the other hand, faster sweep rates can convolute processes.

The response obtained (voltammogram) usually presents three main shapes: an increase in the current with a peak, followed by a decrease. This is a limited process due to analyte kinetics. To overcome this problem, a stirrer can be placed to ensure the analyte reaches the electrode’s surface. This changes the voltammogram to an “S” shape. Usually, the range of potential is assessed in a triangular form known as cyclic voltammetry [63]. This form of voltammetry gives the “duck shape” voltammogram. The last one is the irreversible process, which is usually a straight line. An example of API quantification is Kassa et al., who, through voltammetry, conducted an evaluation of acetaminophen in commercial formulations, and they were able to detect that all brands had the correct amount of API in the formulation as stated on the box [64].

Amperometric measurements involve applying a voltage to an electrode and then measuring the resulting current. This process, like voltammetry, has a great influence on the diffusion layer. As mentioned above, when the potential is applied, it causes a process to occur that can be the reduction or oxidation of an analyte. The process occurs from the electrode’s surface to the solution’s bulk, which leads to a peak and decrease in the current. This process has two types of current, cathodic and faradaic. The cathodic current happens due to the ions’ reorganization in the solution; the faradaic current is from the process (the desired one for evaluation). It is commonly used to assess electrochemical processes, surface modification, or biosensing [65]. Munoz et al. used a reduced graphene electrode for sulfanilamide detection. This was conducted through amperometry in a batch injection analysis (BIA). They were able to detect sulfanilamide in biological samples as well as environmental samples [66].

Lastly, impedance measurement, commonly referred to as Electrochemical Impedance Spectroscopy (EIS), evaluates the system’s resistance across different frequencies. In this technique, an alternating current (AC) or voltage is applied to perturb the system, and the resulting response provides information about the equivalent electrical circuit and the system’s mechanics [67]. EIS is considered a more complex method because the choice of probe and solution conditions must be carefully controlled. Variations in the electrode surface can significantly affect impedance responses, influencing data interpretation and requiring specialized knowledge. Song et al. developed an aptasensor for detecting oxytetracycline [68]. In their approach, a gold electrode was coated with a nanocomposite of iron oxide and mesoporous carbon, followed by immobilization of a specific aptamer for the antibiotic. Using impedance measurements, they successfully detected oxytetracycline in milk, achieving a reported limit of detection of 0.5 pg/mL.

For voltammetric detection, bare metal electrodes, carbon electrodes, and screen-printing electrodes are common. In addition to the bare electrodes, the carbon materials allow surface modifications for target affinity [59]. Although a complete voltammogram can be recorded, more data does not necessarily translate into more meaningful information, emphasizing the importance of focused data analysis [69]. Voltammetric e-tongues are typically employed for food or qualitative analysis, handling liquid samples such as wine and more viscous samples like honey or oil. These systems generally use between two and eight working electrodes. Metal electrodes support techniques such as cyclic voltammetry, square-wave voltammetry, and differential pulse voltammetry [59].

**Figure 6:**
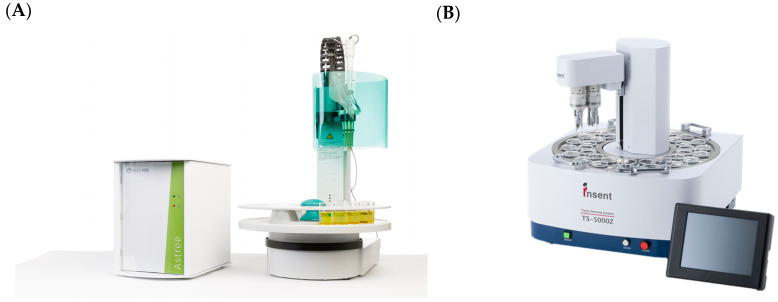
Picture of (**A**) ASTREE electronic tongue from Alpha MOS, Toulouse, France, and (**B**) TS-5000Z taste-sensing system from InSent, Atsugi-Shi, Japan.

The potentiometric sensors were the first type developed [41] and remain the most widely used among commercial options, represented in Figure 6 such as ASTREE (Alpha MOS) [70] and TS-5000Z/TS-6000A (Intelligent Sensor Technology, Inc.) [71], among other options. These sensors can work mainly in three forms: arrays of redundant sensors, arrays of independent sensors, and arrays of cross-sensitivity sensors. As the name suggests, the first one consists of multiple identical sensors to evaluate a faulty sensor or to detect problems with the system; one final option is measuring the concentration gradient when the sensors are in tandem. The second one is for targeting ions, DNA/RNA, or antibodies. It is specific and may experience issues with interference from complex matrices; it is advised to avoid possible contamination during analysis. The last one, represented by commercial E-tongues, is not a sensor with a tailor-made membrane but rather a general membrane, in the case of cross-selective sensors, or a membrane for a specific taste, such as bitter, enabling a complete sample analysis [69].

**Figure 7:**
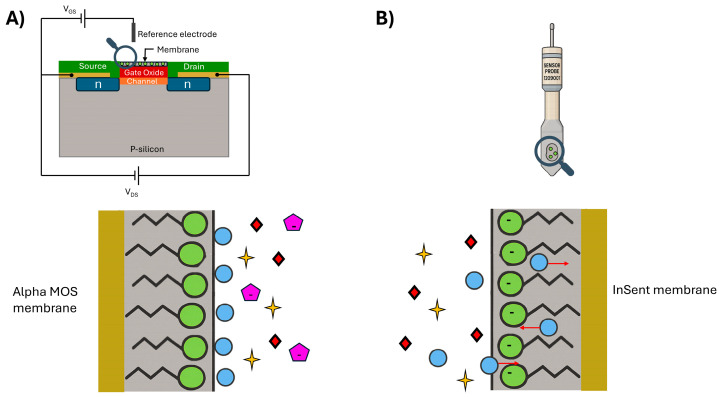
The schematic representation of the membrane’s functionality. (**A**) Alpha MOS ASTREE membrane, and (**B**) InSent TS-5000Z membrane. The different shapes in the figure represents different molecules in the solutuion. The image was made with the aid of ChatGPT-5, OpenAI, 2025.

In the case of InSent equipment, the membranes are composed of lipids and plasticizers and are tailored to interact with specific molecules of each flavor. It is called an artificial lipid-based membrane. The idea of this membrane is to mimic the lipid bilayer [72] that constitutes the taste buds in the papilla [53]. These lipids consist of hydrophilic and hydrophobic parts that can recognize bitter compounds, which are usually hydrophobic, and hydrophilic compounds such as salts and sugars. The recognition of the molecules consisting of the charged part of the lipid layer is exposed to the solution and creates an electrical double layer. Then the ions can interact with the charged part of the membrane (screening effect) or interact with the lipidic part (adsorption). The change in the membrane’s potential is measured to give the taste analysis [53]. This is represented in Figure 7A. More detailed information about the functionality of each piece of equipment can be found in Table 2.

Multiple electrodes are used for flavors of high industrial relevance, such as sweetness and bitterness [73]. Before use, these membranes must be conditioned in a proper solution to be converted from a hydrophobic to a hydrophilic state. The lipids have a polar or charged region that becomes exposed to the solution and will interact with the targeted molecules.

ASTREE equipment employs an Ion-Sensitive Field-Effect Transistor (ISFET) [74], constituting a source, drain, and gate, to complete the system and the reference electrode.

The FET sensor works through the current flow that goes from the source to the drain. This causes a change in the gate’s potential, generating a signal (Figure 5B). This gate has an insulator or membranes on it [75], leading to different types of FET sensors. In the case of ASTREE, there is an ion-selective membrane on the gate of the ISFET sensor. This type of membrane not only interacts with pH and ions but also with chemical structures in molecules, making it possible to differentiate patterns or flavors [75,76].

Although the commercial e-tongue has great importance, tailormade options are also available [77]. In Table 3, other e-tongues that are made for a specific problem are presented. They are laboratory prototypes and, as the commercial ones are only potentiometric, they explore other electrochemical techniques and membranes other than lipid/polymer constitution.

These commercial e-tongues are distributed worldwide. However, they are still premium equipment that requires specialized handling for the mentioned brands, Alpha MOS and InSent. The first company reports that they have sold more than 1000 units of equipment but does not specify how many of each. For InSent, the company reports having sold more than 600 units to major pharmaceutical companies and food chains [42].

### 3.2. Sample and Data Analysis

These sophisticated sensors require careful handing during sample preparation. As good practice, samples must be soluble in water, mimicking the usual consumption of medicine and food. Solid samples that are not fully soluble may contain particles that could adhere to or interact with the sensor membrane; therefore, filtration is mandatory. Solution verification is an addition to sample preparation, determining whether the API of the desired molecule is present in the solution. This could be performed by UV-VIS or HPLC for solution quantification [85,86]. Moreover, this information is important for data processing. Khaydukova et al. developed an e-tongue that was able to perform an online measurement to assess a dissolution test, also using a UV-VIS reference method [87]. This evaluation was conducted for ibuprofen and quinine hydrochloride formulations. When comparing a reference method to an inline e-tongue, the results showed promising performance.

The use of e-tongues for food, such as tomato [88], cocoa [89], cheese, beverages such as coffee and tea [90], wine [91], beer [92], API [35,80,86,93,94,95], and forensic applications [96] has been reported in the literature.

Commercial e-tongues generally operate within a pH range of 2 to 8 (or up to 10) and are incompatible with most organic solvents. Ethanol is one of the few exceptions and can typically be used at concentrations of up to 50%. Moreover, the compatibility of lipophilic substances depends on the specific membrane used in the equipment. Another limitation of the e-tongue is the restricted number of samples it can process and the lengthy time required to complete an entire sample carousel. This process can be time-consuming and demands a trained operator for both sample preparation and data analysis.

As mentioned above, e-tongues usually have multiple sensors to sense all tastes. Numerous electrodes generate multiple individual responses. Therefore, it is necessary to perform multivariate analyses on these data to obtain a complete response. One of the primary methods used in data processing is PCA (principal component analysis), a non-supervised pattern recognition (clusterization). It is a statistical tool that reduces many independent variables to a smaller set. The technique consists of linearly condensing the data into multiple variables and describing the original data set. It means that, although mathematical manipulation reduces the dimensionality from *n* dimensions to two or three dimensions, represented in 2D and 3D graphs that are easier to understand, it reduces the dimensionality from higher- to lower-dimensional data [97,98,99]. This is performed with a matrix to generate Eigenvalues, linearly uncorrelated orthogonal vectors. It mainly consists of the X variance and does not model Y. PCA reduces noise and redundant information, making it easier to analyze the data. However, due to its plainness, it may lead to a loss of signal representativeness [100].

Another option is PLS (partial least squares), which works similarly to PCA, using vectors to best represent X and Y, describing a linear relationship with the set of data/variables, and performing regression and classification. The main distinction between PCA and PLS lies in their purpose and approach: PLS is a supervised method primarily used for calibration, while PCA is an unsupervised technique focused on dimensionality reduction, which can aid in data clustering. PLS uses the Y in addition to X [101] for modeling Y. It evaluates the correlation between a prediction and a response, as PLS also works in dimensionality reduction [102] and is a supervised pattern recognition method. It works well with noisy, incomplete data sets and can handle multicollinear data [100].

KNN (K-nearest neighbors) is a non-parametric method based on the distance between two objects. It is a machine learning algorithm that can be used for classification and regression as PCA and PLS are used, respectively. The distance between two points is usually calculated using Euclidian distance; however, it could also use Manhattan, Minkowsk, and Hamming distances [103].

SIMCA (soft independent modeling of class analogy) is another option for machine learning for group classification. In this case, there is a parametric evaluation as it uses the principal components and dimensionality reduction for the classification of each group [104].

An example of the two methods mentioned above is where vegetable oils are characterized through e-tongue and e-nose and grouped by machine learning methods such as KNN, SIMCA, and other methods to correctly classify in virgin and olive oils [98].

Another helpful method is HCA (hierarchical clustering analysis). This unsupervised analysis generates clusters, through a distance matrix, which can be Euclidean, Manhattan, or another type. The information is divided into clusters, and the more similar they are, the closer they are placed together. These clusters are represented in dendrograms, like a tree, showing which samples are more likely to be of the same type [105]. The dendrogram shows the distance between the clusters, and they will be linked until they are one whole cluster. The information can be filtered, and the dendrogram can be “cut short”.

There is more to data treatment. LDA (Latent Dirichlet Allocation) is one option that recognizes data patterns. This treatment generates new linear synthetic data that best describes the differences between the groups [106].

Lastly, DFA (discriminant function analysis) classifies the individuals and can predict new samples based on their proximity to each group. Another option is nonlinear methods, such as SNE (Stochastic Neighbor Embedding) and t-SNE (t-Distributed Stochastic Neighbor Embedding) [107]. The SNE method consists of reducing the high-dimensional distance using Euclidean distance. The proximity between the data points is preserved, and they are placed together in the representation. It also depends on the perplexity, which ranges from 5 to 50, and can be described as the measure of effective neighbors in the representation. In the optimization phase, Gaussian noise is added to help form the map and is reduced throughout the process. The other option, t-SNE, comes with a t-Student distribution instead of Gaussian noise, resolving the “crowding problem” that may result from Gaussian noise, and a symmetrized version of SNE that has simpler formulas. t-SNE can handle large datasets and can reduce and separate the data into smaller groups [108]. However, it needs to be noted that it is a nonlinear method.

It is also possible to use other analysis forms, like ANN (artificial neural network), CNN (convolutional neural network), and AI (artificial intelligence). It is possible to interpret these data optimally to predict responses for new analyses and molecules [92]. An example of this is provided by Pannone et al., who report the use of KNN for e-tongues. In their work, they were able to create an e-tongue that can distinguish between two brands of soda and adulterated milk due to a KNN model that learned patterns for each individual sample in minute detail, which would have gone undetected by human tasters [109]. This approach minimizes the ISFET sensor drift due to model learning. Extending this idea to AI could yield an ever-learning model; for the pharmaceutical industry, this could lead to a model that could predict better formulation for taste masking, food safety, environmental sample evaluation [110]. Creating a personalized model could help with sensor drift, data bank creation, and data processing, and even help with sensor development. In this sense, AI can enhance e-tongues by automatically extracting and processing the acquired data and providing a response using machine learning and deep learning models in real time. Moreover, this could lead to better pattern recognition and precise responses from complex models. It could also integrate with other sensory analyses, such as smell, for a more complete evaluation. Additionally, AI can support predictive models for new formulations, accelerating the process and reducing the need for taste test trials. Overall, AI has the potential to increase analytical precision, reduce costs, and streamline decision-making in pharmaceutical taste evaluation.

As for an electronic tongue, it still has limitations compared to an actual tongue. As an instrument, it is objective; therefore, it does not have the subjective or bias of the taster, but it cannot understand the complexity of the matrix. Since it can only detect the five basic tastes [106], a real human tester is still required. The e-tongue can perform analyses in as little as 2 or 3 min, which can be robust for industrial production or R&D. On the other hand, it is still limited by data processing, and multivariate analyses, such as PCA, are not easily interpretable by all users. Radar graphs are also used to evaluate changes in each sensor throughout the analysis. As the name states, it is a circular graph, like a radar, where there is a sensor in each axis, and each line represents a measurement. With the change in the composition or concentration, it is possible to see a variation in the response lines. Another option is Euclidean distance evaluation between a blank or reference and a desired sample; it is another suitable alternative for data evaluation.

The e-tongue allows integration with other equipment, does not experience fatigue or require approval by ethical committees, and can reduce costs associated with human taste testing trials. No matter how promising an e-tongue may be, it still has limitations in terms of acquisition cost and commercial availability; therefore, only a few types of e-tongues exist. The sensor membranes can be very limited; some samples cannot be analyzed or need extensive preparation to be analyzed. The sensors require regular calibration, and incorrect use may lead to the loss of a sensor. The equipment is susceptible to external interference and, therefore, can be vulnerable to measurement errors [111,112].

## 4. Assay Nature

Analysis of new compounds or formulations requires validation of taste; however, regarding pharmaceutical formulations, ingestion is not possible. Therefore, robust analysis should be conducted.

**Figure 8:**
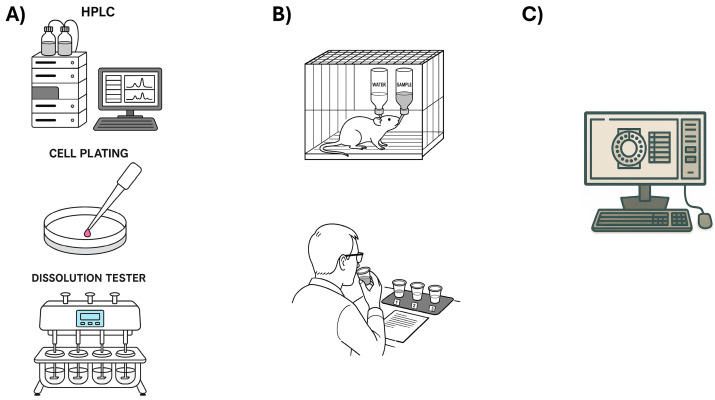
(**A**) Representation of in vitro techniques: liquid chromatography, cell assays, and dissolution, respectively. (**B**) Representation of rat palatability test and human panel. (**C**) Representation of computer processing using machine learning. The image was made with the aid of ChatGPT-5, OpenAI, 2025.

This analysis can be conducted in vitro, in vivo, or in silico. In the context on this review, in vitro techniques include taste masking such as the electronic tongue [113,114] for flavor, and UV [115,116], HPLC [117,118], and dissolution methods [119,120,121] (specific equipment for dissolving formulations with an oral residence period, such as OD-Mate, ODT-101, and TRI-CORPTESTER) for quantification, as well as rheology and viscosity measurements [122] for liquid formulations. For in vivo studies, subjects can include rats [123,124], frogs [125,126], other animals, and human panels [123,127,128,129]. Moreover, in silico analysis consists mainly of predictive methods, such as machine learning [130], ANN [10], and various prediction codes already available in the literature that are generated from data banks, such as BitterDB [131,132], BitterSweet [133,134], and FlavorDB [135]. A schematic representation of each analysis type can is shown in Figure 8.

### 4.1. In Vitro Analysis

In in vitro techniques, the main evaluation is taste. The flavor mainly consists of the API; therefore, quantification of the API is closely related to the acceptance of a drug formulation. In these cases, the release of the API in saliva-like media is highly relevant. Dissolution and disintegration methods and equipment can perform quick analyses in small volumes to mimic the residence time of medicine in the mouth. As mentioned above, specific equipment exists for dissolution, mimicking the dissolution of ODTs in saliva. The most common equipment includes OD-Mate [136], ODT-101 [137], and TRI-CORPTESTER [138]. Compared to a common dissolution, instead of buffered media, it utilizes saliva and smaller volumes [119,120,121], such as 20 mL [139]. Nevertheless, it is important to note that this type of dissolution aids taste evaluation, but it does not replace a human panel or e-tongue [140]. It can relate taste testing to concentration to provide more accurate information on the minimal concentration detectable and mimic the time frame that a formulation may reside in the oral cavity.

In tandem, API release can be quantified using UV probes in real time, or samples can be retrieved and quantified through HPLC and UV-VIS spectrophotometry [115]. API quantification in the solution makes it possible to infer whether the taste is perceived. This is because each molecule has a minimal concentration required to be detected by the taste buds on the tongue.

**Figure 9:**
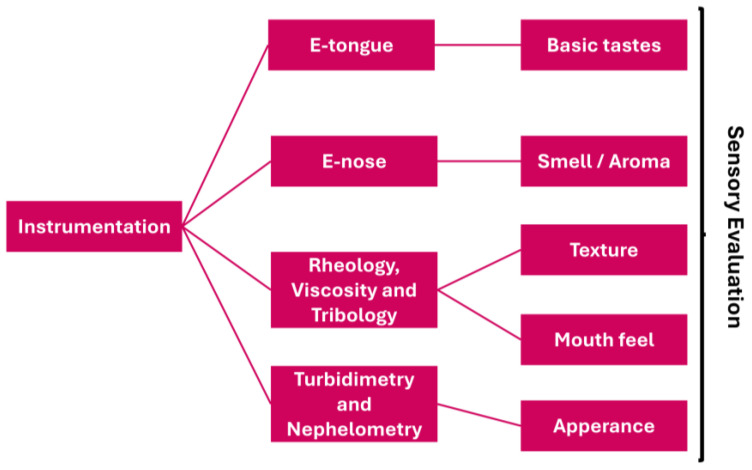
Schematic representation of complementary instrumental analyses for the sensory evaluation of pharmaceutical formulations.

Additionally, sensory analysis extends beyond taste, encompassing texture, color, appearance, and smell. Texture can be evaluated using rheology and texture analysis, which measure viscosity, palatability, and mouthfeel. Tribology provides insights into lubrication and friction. Texture analysis is also helpful in detecting precipitation in solutions or changes over time that may compromise stability. For solid formulations [141], friability and hardness tests can be applied. Appearance can be assessed through turbidimetry and nephelometry, which measure particle dispersion and solubility in suspensions [142]. Gloss measurement is another tool that is helpful in evaluating tablet uniformity and release [143]. A summary is provided in Figure 9.

Another In Vitro analysis uses Chinese hamster ovary (CHO), an epithelial cell line often used in biological, medical, and biotechnology research [144,145]. These cells are easily genetically manipulated; therefore, it is possible for them to express human SGLT1 (hSGLT1), a sodium-glucose cotransporter 1. In humans, hSGLT1 is linked to glucose absorption in the intestine [146]. These cells respond to glucose concentrations, making it possible to test sweet formulations and assess the sweetness. On the same note, human gastric parietal tumor (HGT-1) cells express TAR2R receptors (bitter receptors), which can be measured through fluorescence [147]. Although the signal-to-noise ratio at lower concentrations may be high, it is a good alternative for taste masking or evaluation of new drugs [148]. A summarized representation of the techniques described above can be found in Figure 8A.

### 4.2. In Vivo Analysis

For In Vivo studies, frogs are a suitable option due to their higher expression of TAS2Rs (up to 10 times more than humans) [145]; therefore, they are more capable of sensing bitter taste than humans, making them a suitable model for bitter API taste masking. However, the method utilizes the frog’s nerves, not the whole animal. The analysis is performed by exposing the nerve to the solution and evaluating changes in the nerve potential.

Rodents are another option for liquid and dissolved formulations. The most commonly used rodent is the rat [146]. The total volume of the solution licked is used for assessment. The study is conducted by comparing, within a defined time frame, the total number of licks and the volume of the formulation consumed relative to standard water. If the rodent increases licking and volume, this indicates interest, suggesting that the formulation is “likable”; conversely, if the volume is smaller than that of plain water, the formulation is undesirable or unpalatable [147]. Other animals, like dogs, rely more on smell than taste, making pharmaceutical taste testing less suitable [148].

Lastly, and more importantly, is the human panel. For the human panel, the evaluation considers the flavor (salty, bitter, sweet, umami, and sour), its intensity and residual taste, as well as aroma and texture (delicate, thick, viscous, and rough) [149]. Taste testing is usually conducted with adults; however, for pediatric formulations, it is recommended that children participate in the taste testing [150], although limitations remain. The evaluation is conducted using a scale that may be numeric or hedonic (with expressions/drawings) and ranges from unpalatable to flavorful. All human panels must be approved by the country’s regulatory agency conducting the test.

Additionally, another option for human panels is the TaStation [149,150]. This automated system consists of a 96-well plate mounted on an automated movable base, with a pipette that draws 200 µL. The interface knows the solution placement and concentration in each well. The subject is first given the training solution with an increasing concentration of sugar or salt in order to become familiar with the response interface. Then, the samples are randomly given to the subject for evaluation. The advantage of this system is that, with small sample volumes, testing API solutions would be under the prescribed posology. Moreover, the subject could evaluate more samples, thereby requiring fewer subjects [151]. A representation of animal testing and the human panel can be seen in Figure 8B.

### 4.3. In Silico Analysis

A new option to support the above is in silico, where through machine learning and other predictive methods, molecules, interactions, or, in this review’s case, tastes of new API, as well as suggestions for taste masking of new formulations [152], serves as an ally when taste is discussed (Figure 8C). An available option is the open data banks. The ones available online are BitterDB, FlavorDB, and BitterSweet. Their code and the training materials are available. In the case of BitterDB and FlavorDB, there is a website with information for each molecule and the possible receptors to which it responds [131]. Moreover, BitterSweet accepts SMILES and predicts whether the molecule is bitter or sweet [134]. Another option is Quantitative Structure-Activity Relationships (QSAR), a chemometric tool, often used for API interaction at a molecular level. QSAR models evaluate physical-chemical relationships with biological activity [153], using Leave-N-out and y-randomization.

Table 4 presents a data collection with In Vivo and In Vitro studies, summarizing the number of publications discussed above. All information was obtained from Web of Science.

## 5. Taste Masking

An outstanding alternative is the use of an electronic tongue (e-tongue) to evaluate taste masking in solutions, suspensions, orally disintegrating granules (ODGs), and orally disintegrating tablets (ODTs). With the growing demand for orally dispersible dosage forms, due to their ease of ingestion and faster absorption, ensuring high palatability is essential to achieve a competitive advantage over conventional tablets and capsules. However, these formulations require effective taste masking strategies to be acceptable to patients, as the unpleasant taste of active pharmaceutical ingredients (APIs), often bitter, must be attenuated or masked [154,155]. This is crucial for improving the overall sensory profile of medicines and ensuring patient compliance, particularly in pediatric and geriatric populations, where poor palatability is a significant cause of non-adherence. Additionally, bitterness masking plays an important role because bitter taste aversion is an evolutionary mechanism to avoid the ingestion of toxins [156,157].

**Figure 10:**
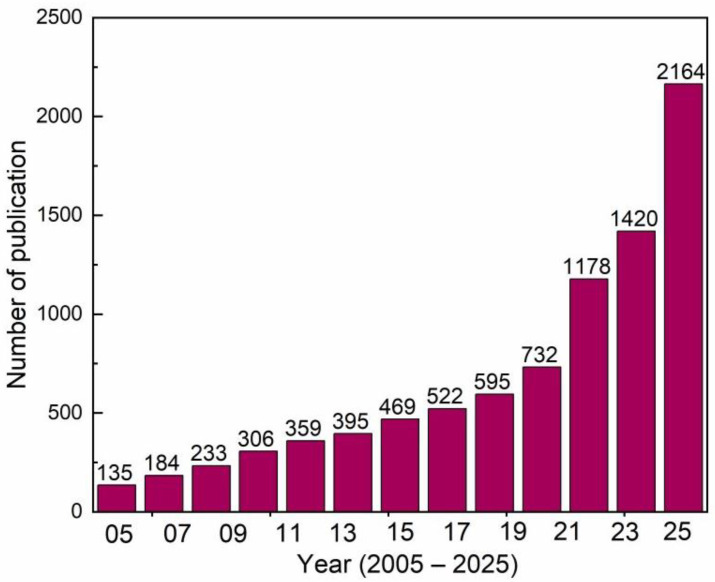
Number of papers published in the last 24 years. The information was extracted from the Web of Science using the keywords “electronic tongue” or “e-tongue” and “taste masking” or “masking efficiency”. The data were obtained up to December 2025.

In another survey from Web of Science, the first published paper containing the word taste masking is from 1946 and has gained interest over time. In this survey, reports with the keywords “electronic tongue” or “e-tongue” and “taste masking” or “masking efficiency” have been collected since 2002, and the last 24 years are summarized in Figure 10. It can be seen that there has been an increase in the trend over the last 10 years.

The primary forms of taste masking are flavor agents, bitterness inhibitors, cyclodextrins, ion exchange resins, solid dispersion, polymer coating, prodrug, microcapsules, liposomes, and nanoemulsions [158,159]. A commonly used technique is a flavoring agent for solutions, suspensions, and orally dispersible forms. These flavor agents can be sweeteners, flavorings, and aromatic agents, which are summarized in Table 5 and Table 6. Flavor agents may vary with world region. Due to their elevated sweetening power, such as mannitol, sucralose, cyclamate, and neotame, they are used for taste masking. It is known that sweets can attenuate bitterness. However, some have laxative action as an adverse effect or present an unpleasant aftertaste at high quantities. It is important to mention that sugars should be dosed with caution due to the sweeteners’ acceptable daily intake (ADI). Moreover, there are reports in the literature that they may present adverse effects, such as unpleasant metallic or bitter taste, and can show antagonist activity on bitter receptors [155,159,160,161]. Zheng et al. developed a 3D printed formulation of levetiracetam instant dissolving tablets [35]. In this work, they evaluated not only sucralose as a sweetening agent but also spearmint as a flavoring. This assessment was performed using an e-tongue and a human panel, showing how the e-tongue can assist the palatability test.

Another solution is to increase viscosity with gums, polymers, or carbohydrates. In a more viscous medium, the mobility of drug molecules is lower. Viscous solutions slow their diffusion from the dosage form to the taste buds, decreasing the immediate perception of bitterness or other unpleasant tastes. High viscosity can create a thicker matrix around the drug particles, forming a physical barrier that limits the interaction between the API and taste receptors. By slowing dissolution and release in the oral cavity, the drug is less available in saliva for direct contact with the tongue. This ensures that most of the drug is swallowed before significant taste perception occurs. Also, increased viscosity provides a “coating” or fuller mouthfeel, which can distract or partially mask unpleasant sensations, making the formulation more acceptable [158]. Bo et al. proposed an evaluation for masking the bitter taste of brivaracetam with cyclodextrins, xanthan gum (polymer), ion exchange resin, and bitter inhibitor. In this case, it was discussed that the increase in viscosity would help hinder API diffusion to the bitter receptors [162].

Cyclodextrins (CDs) can be another choice when the other options above are insufficient; the cage-like structures are capable of solubilizing molecules in solutions due to their toroidal structure, with a hydrophobic interior and hydrophilic exterior, also enabling the trapping of APIs with unpleasant taste [163]. CDs can also be used in solid formulations for stability, dissolution, bioavailability, and other purposes [164]. Busu et al. demonstrated that β-cyclodextrin could make prednisolone a BCS class II soluble and a mouth-dissolving formulation with masked taste. The study concludes that the formulation could be a good alternative for pediatric and geriatric patients with difficulties using conventional solid formulations [165]. Uekemaka et al. showed that β-cyclodextrins could reduce the bitterness of antihistaminic APIs. The assessment was performed by evaluating different forms of cyclodextrins; in this case, the e-tongue determined that β-cyclodextrin and 2-hydroxipropil-β-cyclodextrin gave the best masking [166].

When flavoring agents are not enough, bitter inhibitors can be an alternative. Bitter blockers work by inhibiting the bitter taste pathway; thus, the API’s taste goes unnoticed, and they can act on the initial taste or aftertaste [154,155]. Nonetheless, its effectiveness is still limited as it does not completely inhibit all bitter receptors or sufficiently reduce bitterness for the API to be tolerable. Another option is the usage of amino acids, such as alanine and taurine, which can combine with bitter APIs and reduce the unpleasant taste.

A well-known form of taste masking is sugar coating, which is now rarely used. As the name suggests, it is a sugar coating around the tablet. It increases tablet volume and has some design limitations. Amat et al. studied sugar coatings for cyclophosphamide and its benefits compared to its corresponding liquid solution, which is better for API stability and does not involve water [167,168].

Ion exchange resins could be another alternative. It works by complexing the distasteful API with a resin. This new complex is insoluble at mouth pH; therefore, it has no taste. When it reaches the acidic environment of the stomach, the API releases itself from the complex (swapping places with H+), enters the gastric solution, and can be absorbed [165]. Jantrawut et al. used ion exchange resin, in this case Amberlite IRP-69 and Dowex-50, for masking the taste of Nizatidine. The drug/polymer ratio could be evaluated with different proportions with an e-tongue and a human panel [169].

Polymer coating can be a good alternative to taste masking. The API granules, pellets, or other forms are coated with a polymer, hindering the API from interacting the taste buds in the mouth. Afterwards, it is digested in gastric or enteric fluids, and the dissolution process can occur. The polymers include metacrylates, polyethylene glycol–polyvinyl alcohol, and chitosan [170,171]. An example of the use of polymers is in ondansetron ODTs. Yeole et al. evaluated the aminoalkyl methacrylate copolymer with the API at different ratios to obtain the best taste masking and without interfering with the rapid-disintegrating release [170]. It is essential to study the coat thickness for an even and uniform coating; otherwise, it could have the opposite effect, or a thick coat could produce a gritty sensation or lead to uneven coating. In this case, the production process, including the mask component, is also important for the best masking efficiency.

Chemical modification includes prodrugs, API salts, and pH transformation. The API molecule undergoes a structural change for a prodrug, and a functional group is usually added. This functional group undergoes cleavage route that can regenerate the original API [172]. This structural modification can also reduce the unpleasant taste. Through a computational approach, Karaman showed that there are certain structures that do not interact with the bitter receptors and have the potential for sustained release [172]. Karaman also reported on the antibacterial drugs chloramphenicol and clindamycin [173]. In both cases, prodrugs are used: palmitate chloramphenicol and palmitate clindamycin. They are tasteless when compared with the active molecules.

The same applies for pH-dependent structures, which can be salts or polymer coatings. This is an option for taste masking, where the API is usually insoluble in the mouth (neutral pH); therefore, the taste is not perceived, and it becomes soluble in the lower gastric pH environment [174]. Similarly, pH-dependent API salts work in the same manner. Changing the cation or anion in the structure alters the solubility, enabling the compound to be soluble only in more acidic media, or to form a more soluble overall variant, thereby changing the pharmaceutical presentation. Another pH-dependent approach uses polymers for taste masking that are pH-dependent and have a specific pH release, as presented by Al-Kasmi et al. for a new formulation of paracetamol pellets with Eudragit E (methacrylate polymer) [175].

**Figure 11:**
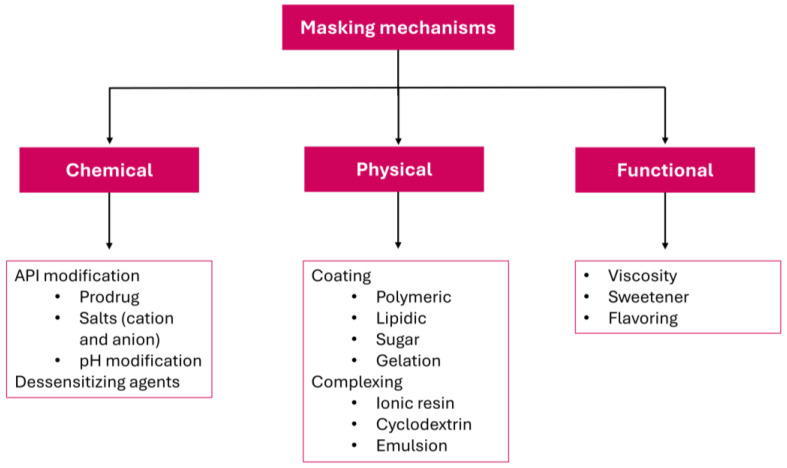
Diagram of masking mechanisms described above.

Lastly, desensitizing agents can be a good alternative. They work by interfering with the taste bud, reducing or eliminating bitter taste signaling [158,176]. These can include organoleptic agents such as menthol, which not only imparts a distinct taste but also provides a cooling effect that helps reduce bitterness. Similar effects are observed with eucalyptus oil, fenchone, borneol, and isoborneol. The properties of the active ingredient should be evaluated first to determine the most suitable taste-masking technology. For ionic molecules, ion-exchange resins may be effective, while for hydrophobic drugs, cyclodextrins could be a viable option [154]. A concise representation of masking forms is shown in Figure 11.

## 6. Key Findings and Future Perspectives

The unmatched capabilities of the e-tongue—the capacity for taste-sensing compounds that may be viable only in micro-dosing, testing multiple samples for taste masking, and analyzing complex samples—without the need for approval to conduct human panel analysis and do not present fatigue as the real tongue does are of paramount importance. Furthermore, the reduced time required to perform analysis, with simple sample preparation, can also be an alternative to narrow down the options for a human panel. It can be helpful not only for the pharmaceutical industry but also for the food industry for taste evaluation. It can be adapted to sense all tastes, like with Astree, or be more focused on bitterness and sweetness, such as with InSent. However, it is possible to tailor a sensor to the designed target.

It is important to note that commercial e-tongues still have several limitations. The equipment lacks objectivity and cannot identify the specific flavor characteristics of each sample as humans can; it does not perform taste screening but only comparative analysis. Additionally, it is limited by the number of samples that can be analyzed per batch and the time required to complete a full analysis. In terms of sample types, it can only handle soluble solutions, while suspensions and oily samples cannot be processed. Operating the equipment requires a trained analyst for both sample preparation and data interpretation. Furthermore, challenges remain regarding data reproducibility and normalization. For pharmaceutical formulation analysis, reports are still scarce. Conversely, in academic settings, e-tongues tend to be more customizable, and the knowledge surrounding their use remains largely confined to research environments.

Nevertheless, it is worth mentioning that, as equipment, it needs regulations and validation from the pharmaceutical industry. Nowadays, it is used to support research and development in new formulation development projects. It is not present in the product life cycle. Validation for both types of e-tongues is reported in the literature in accordance with ICH Q2 guidelines. It is worth noting that more theoretical information is available for InSent equipment, as details about its sensors are well-documented. Standardization is also critical; in routine applications, all analyses must follow established guidelines and deliver reproducible results. Since e-tongues are relatively new for routine use, their operation is not yet widely understood. Additionally, they represent a significant investment due to their high cost and the initial need for specialized personnel. Challenges related to reproducibility and normalization remain, and for pharmaceutical formulation analysis, published reports are still limited. Conversely, in academic settings, e-tongues tend to be more customizable, and the knowledge surrounding their use is largely retained within research environments.

The first electronic tongue may have been invented in the 1990s; however, it is still developing, and technology has the potential to grow with the integration of AI, machine learning, and the possibility of pattern recognition. In the case of pharmaceutical applications, it could be designed to enable taste masking and help with the design of new molecules.

## Figures and Tables

**Table 1:** Summarized electronic tongue storyline through the decades.

Year	Development	References
1940s	Electronic brain development	[37]
1960s	Wilkins and Hartmann pioneered the e-nose	[13]
1980s	First e-nose, and Toko filed the first patent for a taste sensor	[39]
1990s	The definition of e-tongue, collaboration between Kyushu University and Anritsu Company for SA401 and later SA402 (future Insent). Filed patents for AlphaMOS e-tongue and Winquist and Hartmann’s voltammetric e-tongue.	[41,42,43,44,45]
2000s	The first commercial electronic tongues appeared for AlphaMOS and InSent.	[42,46]
2010s and 2020s	In the 2010s and 2020s, commercial equipment was enhanced, new models were introduced, and more applications were obtained in pharmaceutical and food industries.	[42]

**Table 2:** Comparison between ASTREE and TS-5000Z.

	ASTREE	TS-5000Z
Analysis	Solution in the equipment beaker	Solution in the equipment beaker
Number of analyses	16 or 48 samples	Up to 14 samples
Sensor technology	ChemFET/ISFET	Potentiometric sensor with a lipid/polymer membrane
Number of sensors	7 sensors	up to 8 sensors
Definition of each sensor/sensor set	The sensors are different from each other; the analysis is performed globally (all sensors must be considered for the evaluation)	The sensors are specific for each taste attribute, and the composition is known. There is a sensor set specifically for pharmaceutical analysis. There is more than one sensor for bitter taste
Aftertaste analysis	No	Yes
Analysis duration	2 min (1 measurement in default condition)	40 min per sample (Cleaning + Reference + Sample)
Molecule type that the sensors can analyze	Ionic *	Ionic. Some neutral samples can also be analyzed, but they should be checked first
Measurement description	When the (Conditionament + Calibration + Diagnostic) CCD is approved, the sample’s reading can be performed. Then, the beakers must be placed as instructed by the software. The sequence should be as follows: the sample, followed by the cleaning solution. A complete sample reading should consist of 6 to 9 readings per sample.	The measurement starts with the sensor in the reference solution, where it was stabilized. It is then moved to the sample solution to take readings, followed by a short cleaning process, and then an aftertaste reading in the reference solution. Finally, the sensor is cleaned thoroughly with an ethanol solution.

* Our research group tested and was able to analyze non-ionic molecules successfully. All the data were obtained from the suppliers.

**Table 3:** Tailormade e-tongues.

Tailormade e-Tongue	Membrane Material	Form of Analysis	Number of Sensors	Reference
Microfluidic electronic tongue for oral cancer	PDMS structure with microwires of SiO_2_, NiO_2_, Al_2_O_3_, and Fe_2_O_3_ functionalized with SiO2NP	Impedance	4	[78,79]
Electronic tongue for taste-masking	PVC, DOS, o-NPOE, KTFPB, KTPClPB, TDMAC, and Ionophores	Potentiometric	9	[80]
Electronic tongue for paracetamol evaluation	Ionophorates, borates, tetraphenylporphirine chlorides, and other salts	Potentiometric	19	[81]
Electronic tongue for phytomedicine application	Lipid membrane with PVC, DOPP, DA, OA, TOMA, and OAm	Potentiometric	8	[82]
Electronic tongue for polyphenols	Layer-by-layer film of a AgNP, phospholipid, and PAH mixture	Impedance	6	[83]
Molecularly imprinted polymer for pharmaceutical e-tongue	p-toluenesulfonic acid and pyrrole	Differential pulse voltammetry	4	[84]

PAH = polynuclear aromatic hydrocarbons, AgNP = silver nanoparticle, SiO_2_NP = silica nanoparticle, PVC= polyvinyl chloride, DOP = dioctyl phosphate, DA = decyl alcohol, OA = oleic acid, OAm = oleyl amine, DOS = bis (2-ethylhexyl) sebacate, o-NPOE = o-nitrophenyl octyl ether, KTFPB=potassium tetrakis [3,5-bis(trifluoromethyl)phenyl]borate, KTPClPB = potassium tetrakis (4-chlorophenyl)borate.

**Table 4:** Number of publications with In Vivo and In Vitro themes, and keywords (December 2025).

In Vitro	Number of Publications
“Electrochemical tongue” and “dissolution”	62
“Electrochemical tongue” and “spectrophotometry”	17
“In Vivo” and “pharmaceutical palatability”	43
“In Vivo” and “pharmaceutical palatability” and “rat”	25
“In Vivo” and “pharmaceutical palatability” and “animals”	55

**Table 5:** Commonly used flavoring agents by drug class.

Drug Class	Flavorings
Antibiotics	Maple, Tutti-Frutti +/Cinnamon, Strawberry +/Vanilla, Orange, Cherry, Banana + Vanilla, Raspberry, Pineapple and Caramel, Grape
Antihistamines	Grape, Cherry, Honey, Wild Cherry, Orange + Peach, Cinnamon, Chocolate, Vanilla, Raspberry and Apricot.
Barbituric	Banana + Pineapple, Banana + Vanilla, Mint, Strawberry, Orange +/Peach, Cinnamon-mint
Decongestants and expectorants	Anise, Clove, Apricot, Cherry, Orange, Orange-lime, Fennel, Strawberry, Raspberry, Pineapple, Strawberry + Mint, Orange + Lime, Cream + Mint + Strawberry, Honey, Honey + Ginger/Orange
Electrolytes	Cherry, Mango, Raspberry, Orange, Wild Cherry, Tutti-Frutti, Wild Strawberry, Grape, Passion fruit, Yellow fruits, Berries, Grape, Guarana

**Table 6:** Commonly used flavoring agents by flavor.

Flavor	Flavoring
Sour	Tutti-frutti, cherry, lime, orange, passion fruit, strawberry, berries
Bitter	Raspberry, glycyrrhiza (licorice), eriodictyon, wild cherries, chocolate, chocolate-mint, walnut, orange, lime, vanilla, cappuccino, coffee, churros, cinnamon, guarana with acai
Sweet	Strawberry, grape, raspberry, vanilla, acacia syrup, tutti-frutti, chocolate, milk
Sour	Glycyrrhiza (licorice) syrup, maple, butterscotch, cinnamon, raspberry, orange, chocolate, milk, pacoca, pistachio, dulce de leche
Metallic	Strawberry, raspberry, cherry, grape, cappuccino, dulce de leche

## Data Availability

Not applicable.
